# Association of Food Intake Quality with Vitamin D in SARS-CoV-2 Positive Patients from Mexico: A Cross-Sectional Study

**DOI:** 10.3390/ijerph18147266

**Published:** 2021-07-07

**Authors:** Guillermo González-Estevez, Francisco Javier Turrubiates-Hernández, Laura Elena Herrera-Jiménez, Gabriela Athziri Sánchez-Zuno, Melva Guadalupe Herrera-Godina, José Francisco Muñoz-Valle

**Affiliations:** 1 Institute of Research in Biomedical Sciences, Department of Medical Clinics, University Center of Health Sciences (CUCS), University of Guadalajara, Edificio Q, 950 Sierra Mojada, Guadalajara 44340, Mexico; guillermo.gonzalezestevez@cucs.udg.mx (G.G.-E.); francisco.turrubiates3337@alumnos.udg.mx (F.J.T.-H.); laura.herrera3967@alumnos.udg.mx (L.E.H.-J.); athziri.sanchez@alumnos.udg.mx (G.A.S.-Z.); melva.herrera@academicos.udg.mx (M.G.H.-G.); 2 COVID-19 Situation Room (Analysis Group), University Center of Health Sciences (CUCS), University of Guadalajara, Guadalajara 44340, Mexico

**Keywords:** vitamin D, SARS-CoV-2, food intake quality, physical activity, COVID-19, coronavirus, calcidiol, micronutrients, Mexico

## Abstract

One of the micronutrients that has attracted the most attention in relation to COVID-19 is vitamin D. Although several factors affect its sufficiency; it has been argued that an optimal diet can ensure the intake of micronutrients with effects on immune response. Therefore, in this work we aimed to evaluate the food intake quality of SARS-CoV-2 positive Mexican patients and some of the common factors related to vitamin D deficiency. We conducted a cross-sectional study in 40 SARS-CoV-2 positive patients. Serum samples and clinical parameters were collected. Micronutrient intake and food intake quality were assessed with a 24-h dietary recall and the Mini-ECCA v.2, respectively. Thirty-eight percent of the sample had a healthy food intake. The median 25(OH)D concentration was 22.7 ng/mL. A considerable insufficient intake of micronutrients with immunomodulatory effects such as vitamin D (*p* < 0.0001), vitamin E (*p* < 0.0001), and zinc (*p* < 0.0001) was shown. Patients with 25(OH)D sufficiency, defined as a concentration >30 ng/mL, had better food intake quality (*p* = 0.02) and an intense physical activity (*p* = 0.03). In conclusion, a better level of food intake quality and intense physical activity are associated with 25(OH)D sufficiency in SARS-CoV-2 positive Mexican patients.

## 1. Introduction

The novel coronavirus identified, as severe acute respiratory syndrome coronavirus 2 (SARS-CoV-2), responsible for coronavirus disease 2019 (COVID-19) continues to infect thousands of people daily worldwide [1,2]. Genetic and environmental factors such as obesity, male gender, older age, black ethnicity, urban residence, and chronic kidney disease, among others, are associated with SARS-CoV-2 predisposition [3]. Likewise, some of these factors have also been associated with the severe prognosis of this disease [4].

Among the prevention strategies for COVID-19, it is proposed that the implementation of recommendations for optimal nutrition is crucial in the clinical course of the disease [5]. In particular, it has been stated that a healthy diet is necessary for the proper functioning of the immune response, since, through a balanced and varied diet, the recommended intake of micronutrients with effects on the immune system can be obtained [6].

Vitamin D is one of the micronutrients that has attracted most attention in COVID-19. Studies that have evaluated the concentration of 25-hydroxyvitamin D (25(OH)D), which corresponds to the main serum biomarker of this vitamin, have reported a high prevalence of 25(OH)D deficiency in patients with COVID-19, which has also been associated with the incidence and course of the disease [7,8]. The importance of vitamin D is due to its potential immunomodulatory effects that may prevent the severity of COVID-19 [9]. However, deficiency of this vitamin is considered a global public health problem where it is estimated that one billion people have deficient concentrations of 25(OH)D, while insufficiency affects ~50% of the world’s population [10].

Due to the geographic location of Mexico, as well as other Latin American countries where the intensity of the sun’s rays is more powerful, vitamin D deficiency should not be frequent; however, several genetic, pathological, and environmental factors affect serum 25(OH)D concentrations [11]. According to a national study, the Mexican population has a high prevalence of insufficient intake of vitamins and minerals [12]. In addition, according to studies of other populations, measures to contain the spread of SARS-CoV-2 have modified lifestyles that include healthy eating and physical activity [13,14,15]. This is of concern, since the Mexican population, as well as other populations in the region, have been strongly affected by the COVID-19 pandemic where, in addition, they could be at risk of insufficiency of immunomodulatory micronutrients without taking into account the limited immunization rates.

Based on the importance of a healthy diet in the immune system and that containment measures may have increased the insufficient intake of micronutrients, in this work, we aimed to evaluate the food intake quality in SARS-CoV-2 positive Mexican patients. Additionally, we analyzed which factors are related to 25(OH)D deficiency in this population. 

In the present study, we describe micronutrient intakes, some of which are significantly lower than the Recommended Dietary Allowance (RDA) for Mexicans. We also classified patients according to their serum 25(OH)D levels and found that a better food intake quality classification is associated with 25(OH)D sufficiency.

## 2. Materials and Methods

### 2.1. Study Design, Settings and Participants

We carried out a cross-sectional study. Subject recruitment was conducted from August to September 2020. The University of Guadalajara (UdeG) set up an open call center for the general population with COVID-19 symptoms. Subjects who contacted the call center were interviewed. Those individuals who reported at least one COVID-19 symptom (fever, dry cough, muscle pain, bone pain, fatigue, diarrhea, etc.), a risk factor (healthcare worker, >60 years old, obesity, hypertension, etc.), and six days or fewer with symptoms were scheduled at the Emerging and Reemerging Diseases Diagnostic Laboratory (LaDEER) for a reverse transcriptase-polymerase chain reaction test (RT-PCR, assay kit: DeCoV19 Kit Triplex Cat. G2L-DeCoV19-MP, Genes2Life, Guanajuato, Mexico) capable of detect the N1, N2, and N3 genes of SARS-CoV-2. We have reported in detail the eligibility criteria for RT-PCR in another study [16].

Individuals with SARS-CoV-2 positivity over 18 years of age were invited to participate in the present study. Those who reported recent vitamin D supplementation, people with malabsorption disease, and pregnant or breastfeeding women were excluded.

### 2.2. Measurements and Data Collection

Once the participation of the patient was confirmed, serum sample was collected following a 12-h overnight fast. Transferrin was analyzed by turbidimetry with the assay kit VITROS^®^ Cat. 6801767 (Ortho Clinical Diagnostic, Raritan, NJ, USA). Ferritin (assay kit: VITROS^®^ Cat. 8356636, Ortho Clinical Diagnostic, Raritan, NJ, USA), D-dimer (assay kit: MAGLUMI^®^ Cat. 130206008M, Shenzhen New Industries Biomedical Engineering, Shenzhen, China), and 25(OH)D (assay kit: LIAISON^®^ Cat. 310600, DiaSorin, Saluggia, Italy) were determined by chemiluminescence. The patients were classified according to their 25(OH)D concentration based on the Endocrine Society criteria: <20 ng/mL, deficiency; 21 to 29 ng/mL, relative insufficiency; >30 ng/mL, sufficiency [17]. All participants completed a survey of sociodemographic data, history of comorbidities, time of sun exposure, and Fitzpatrick skin phototype [18]. We also recorded physical activity using the International Physical Activity Questionnaire (IPAQ) instrument in its “usual week” version [19]. The presence of COVID-19 symptoms (cough, headache, muscle pain, anosmia, ageusia, and fatigue) was taken into account.

We assessed the vital signs and anthropometric data of each individual. The temperature was measured with an infrared thermometer (Yuwell YT-1C, Yuwell-Jiangsu Yuyue Medical Equipment & Supply, Shanghai, China), oxygen saturation was determined with a fingertip pulse oximeter (Sonolife LMT-01, Sonolife, Shanghai, China), and blood pressure was measured with a digital blood pressure monitor (Omron HEM-RML31, Omron Healthcare, Kyoto, Japan). Anthropometric measurements were performed with a portable stadiometer (SECA 213, Medical Measuring Systems and Scales, Hamburg, Germany), digital body weight scale (Beurer BG21, Beurer, Swabia, Germany) and tape measure (Lufkin W606, Lufkin, OH, USA) according to international standards. Body mass index (BMI) was calculated: weight (kg)/height^2^ (m). BMI and waist circumference were classified according to World Health Organization (WHO) parameters [20]. Normal weight, 18.5 to 24.9 kg/m^2^; Overweight, 25 to 29.9 kg/m^2^; Obesity, ≥30 kg/m^2^. Central obesity: waist circumference ≥88 cm in women and ≥102 cm in men.

In the afternoon of the same day or the following day, we conducted the dietary evaluation via video call. We obtained a 24-h dietary recall that was analyzed with the Mexican Food Database (BAM, Spanish acronym) in its 18.1.1 version [21] for determination of nutrient intake. Likewise, the Mini-Survey to Evaluate Food Intake Quality (Mini-ECCA v.2) was applied. This is a tool validated in the Mexican population to determine food intake quality. The Mini-ECCA v.2 consists of 14 items with three or four Likert-scale response options. Based on the score obtained, this instrument has three classifications: healthy food intake; habits in need of improvement; and unhealthy food intake [22]. The RDA’s for the Mexican population were considered (NOM-051-SCFI/SSA1-2010 in its 2020 update, URL: https://www.dof.gob.mx/2020/SEECO/NOM_051.pdf accessed on 15 April 2021). We calculated the percentages of adequacy of dietary intake of micronutrients for each of the patients (nutrient intake × 100/RDA). Subsequently, patients were classified according to the following categories for the Mexican population [23]: <90%, insufficient; 90–110%, adequate; and >110%, excessive. The latter analysis was performed based on the methodology reported by Meza-Meza MR, et al. 2019 [24].

At all times, the personal protective equipment guidelines recommended by the Centers for Disease Control and Prevention were complied with for both patients and healthcare personnel who collaborated in the study.

### 2.3. Statistical Analysis

The analysis was performed in the statistical package IBM SPSS Statistics v 25.0 (Chicago, IL, USA) and GraphPad Prism v 5.0 (San Diego, CA, USA) with a statistical significance level of *p*-value < 0.05. A normality analysis was performed using the Shapiro-Wilk test to determine the distribution of the numerical variables. Numerical variables with normal distribution are described as means ± standard deviation, while those with non-normal distribution are described as medians (percentiles: p5th-p95th). On the other hand, categorical variables are expressed in frequencies and percentages, *n* (%).

Comparison between groups was made according to the distribution or nature of the variables using Student’s *t*-test, Mann-Whitney *U* test, chi-square (*χ^2^*) test or Fisher’s exact test. We performed a correlation analysis using Spearman’s correlation coefficient (*rs*) and a univariate and multivariate logistic regression analysis. The R-squared (*R^2^*) of the models were recorded. Micronutrient intake was adjusted for total energy using the residual method [21].

## 3. Results

### 3.1. Clinical Features of SARS-CoV-2 Positive Patients

A total of 40 SARS-CoV-2 positive patients were included, of which 16 (40%) were male. The mean age was 43.98 years. Most patients (95%) reported COVID-19 symptoms at the time of their diagnosis; cough (65%), headache (62.5%), and muscle pain (57.5%) were the most prevalent. Thirty percent of the patients included had some type of comorbidity, with hypertension being the most common (50%). The average heart rate and temperature of the patients were within the normal ranges. The median 25(OH)D concentration was 22.7 ng/mL. Concerning evaluation of the food intake quality, only 37.5% of the assessed patients had a healthy food intake (Table 1).

### 3.2. Dietary Micronutrient Intake of SARS-CoV-2 Positive Patients

From the 24-h dietary recall, we analyzed the dietary intake of micronutrients. The average intake of vitamin A was 508 μg RE with an adequacy percentage of 89.5% based on the RDA’s for the Mexican population. Concerning dietary intake of vitamin C, there was no significant difference between what was reported by the patients and the RDA’s; however, the adequacy percentage was 133.34%. On the contrary, vitamin D, E, calcium, iron, and zinc intakes were significantly lower compared to the RDA’s with a *p*-value < 0.0001 for all five micronutrients (Table 2).

**Figure 1:**
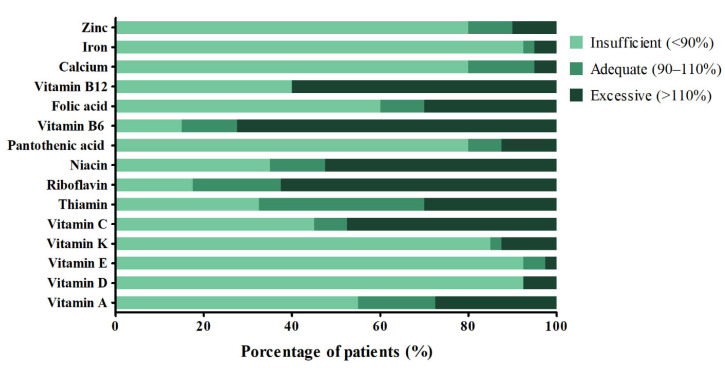
Prevalence of adequate micronutrient intake. The percentage of adequacy of each patient was classified as: <90%, insufficient intake; 90 to 100%, adequate intake; >110%, excessive intake. The figure shows the percentage of patients in each of these categories.

In order to determine the proportion of patients with intakes within the adequacies for the Mexican population, a performed analysis showed that a large part of the sample had an insufficient intake of micronutrients. Only 17.5% of the patients evaluated had sufficient vitamin A intake. Insufficient intake for both vitamin D and vitamin E was found in 92.5%. Likewise, there was a high prevalence of insufficient intake of essential trace elements involved in immune function such as zinc (80%) and iron (92.5%) (Figure 1).

In the correlation analysis, the serum 25(OH)D concentration of SARS-CoV-2 positive patients was not related to any parameter used as a biomarker in COVID-19, such as transferrin, ferritin, and D-dimer (data not shown). On the other hand, dietary vitamin D intake (*rs* = 0.320, *p* = 0.04) and the minutes per day of intense physical activity (*rs* = 0.345, *p* = 0.02) were positively correlated with 25(OH)D serum concentration.

### 3.3. Comparison of Clinical Features and Food Intake Quality of SARS-CoV-2 Positive Patients with 25(OH)D Insufficiency and Sufficiency

To identify which factors might contribute to 25(OH)D sufficiency, a contrast analysis was performed between the groups with 25(OH)D insufficiency (<29 ng/mL, *n* = 32) and sufficiency (>30 ng/mL, *n* = 8). Patients with 25(OH)D sufficiency presented a better food intake quality (*p* = 0.02). Likewise, this group of patients reported a longer time of sun exposure (*p* = 0.01) and minutes per day of intense physical activity (*p* = 0.03). The presence of COVID-19 symptoms was higher in the 25(OH)D insufficient group (*p* = 0.03) (Table 3).

**Figure 2:**
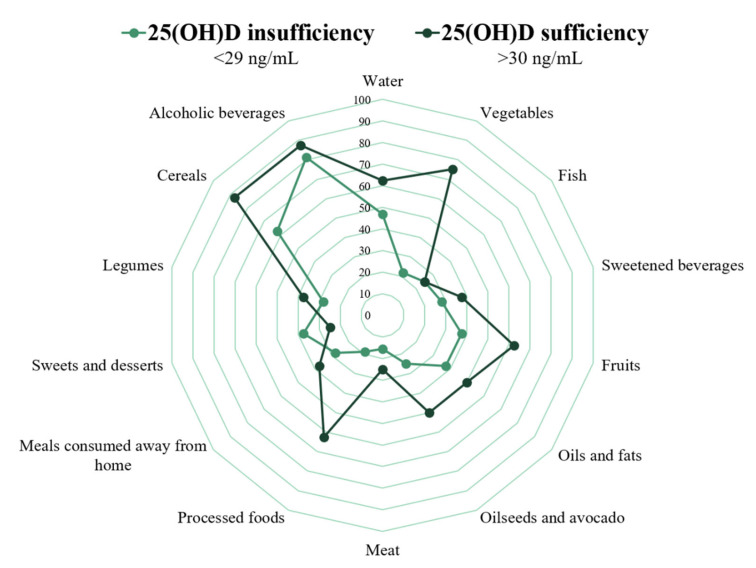
Prevalence of the recommended level in each item on the food intake quality questionnaire in patients with 25(OH)D insufficiency and sufficiency.

Likewise, a spider chart was plotted with the 14 Likert-scale items of the food intake quality questionnaire. The percentage of patients in each group who reported the recommended level in each item was contrasted. In the 25(OH)D sufficiency group there is a higher prevalence of people with a recommended score for vegetable intake (75% vs. 21.9%, *p* = 0.04) (Figure 2).

Finally, in the multivariate logistic regression analysis we found an association between the highest level of food intake quality and 25(OH)D sufficiency (OR = 6.79 [95% CI: 1.18–39.09; *p* = 0.03]). Minutes per day of intense physical activity were also associated with 25(OH)D sufficiency (Table 4).

## 4. Discussion

The present study demonstrates that less than half of the SARS-CoV-2 positive patients from the assessed Mexican population had a healthy food intake. It was also identified that there is an inadequate dietary intake of micronutrients involved in the immune response and a high prevalence of 25(OH)D insufficiency and deficiency in this population group. We also found that a better food intake quality classification and more minutes per day of intense physical activity are associated with 25(OH)D sufficiency.

More than a year after COVID-19 has been classified as a pandemic, as of 31 May 2021, the Johns Hopkins University dashboard reports more than 170.5 million cases and more than 3.5 million deaths from COVID-19 globally [2]. Among the risk factors, comorbidities related to malnutrition have been associated with a higher incidence of SARS-CoV-2 infection and severe disease prognoses [3,4]. Therefore, it has been suggested that the nutritional status of the patients is crucial in the clinical outcome of viral infections since suboptimal micronutrient status can negatively affect the immune response [6].

One of the vitamins that has attracted the most attention in relation to COVID-19 is vitamin D. The importance of a sufficient concentration of this vitamin in relation to COVID-19 is due to its potential immunomodulatory effects such as maintenance of epithelial cell integrity, promotion of antimicrobial peptides, modulation of antigenic presentation by dendritic cells, promotion of anti-inflammatory cytokines, and regulation of renin production [9]. However, a high prevalence of deficient 25(OH)D concentrations in patients hospitalized for COVID-19 has been reported [25,26]. Likewise, vitamin D deficiency has been associated with COVID-19 incidence, hospitalization, and mortality worldwide [7,8].

In theory, the Mexican population should not be vitamin D deficient since the sun’s ultraviolet-B (UV-B) radiation is more powerful in countries located between latitudes ~33° in both hemispheres [17]. However, in the present study, more than half of the individuals evaluated had 25(OH)D insufficiency. This result is consistent with that reported by other research groups [27,28,29,30]. Exposure to UV-B rays from the sun accounts for 80% of the source of vitamin D by inducing its synthesis in the skin. The remaining 20% is obtained through diet [9]. Thus, the purpose of this study was also to evaluate factors associated with 25(OH)D concentration in SARS-CoV-2 positive patients.

According to studies reported by Bracale, et al. [13], and Deschasaux-Tanguy, et al. [14], quarantine has led to changes in dietary habits. In particular, the consumption of fresh foods such as vegetables, fruits, milk, and fish has decreased. Likewise, another study reported by Cicero, et al. [15] shows that diet quality may worsen as a consequence of quarantine. In this last report, although there was an increase in the intake of vegetables, fruits, and fish, there was also an increase in the consumption of sugars, fats, and alcohol. In our study, only 37.5% presented a healthy food intake which meant, according to the instrument used (Mini-ECCA v.2), that the diet of these individuals was characterized by a better intake of water, vegetables, fruits, oilseeds/avocado, lean meat, fish, healthy fats, and legumes, and accompanied by lower consumption of sweetened beverages, sweets and desserts, processed foods, and alcoholic beverages.

In a study by Bogataj Jontez, et al. in healthy individuals [31], there was also a slight worsening in diet quality during quarantine; however, concerning dietary intake of vitamin D, it remained considerably below the RDA’s during five months of quarantine. Although in our study, dietary intake assessment was only performed once, as previously reported in a population-based study by Ramírez-Silva, et al. [12], the dietary intake of vitamin D in the Mexican population is 2.62 ± 1.22 μg; therefore, according to this report, vitamin D intake was estimated to be deficient in the patients evaluated regardless of pandemic containment measures.

In the present study, 92.5% of the patients evaluated did not meet the vitamin D RDA’s. This result is similar to that reported by Dimakopoulos, et al. [32] where 100% of the population evaluated reported an intake below the RDA’s. Additionally, it was identified that the main source of vitamin D through the diet is the intake of fish, meat, and cereals [32]. Likewise, according to Yoo, et al. [33], foods that contribute the most to dietary vitamin D intake include seafood, eggs, dairy, meat products, mushrooms, and grains. In this same study, even though the population evaluated did not exhibit adequate vitamin D intake, increased intake of vitamin D may predict higher 25(OH)D concentrations. The latter result is consistent with the positive correlation between vitamin D intake and 25(OH)D concentration in the general population of our study.

When we classified the individuals in our study by 25(OH)D concentration, we identified that a higher level of food intake quality was associated with 25(OH)D sufficiency. Concerning this analysis, only vegetable intake was statistically different between the two groups; however, the tool used represents only one week’s frequency of healthy food intake and does not cover the intake of all vitamin D-rich foods. Furthermore, taking into account that the half-life of 25(OH)D is approximately 15 days [34], the 24-h dietary recall and the food intake quality questionnaire partially reflect food intake associated with 25(OH)D concentration. We consider that a possible explanation for this association is that individuals who presented a better food intake quality commonly have a balanced and varied diet and a healthy lifestyle.

Concerning a healthy lifestyle, according to Tønnesen, et al. [35], low physical activity, smoking, fast food consumption, and obesity are associated with 25(OH)D deficiency. Our study identified an association between minutes per day of intense physical activity and 25(OH)D sufficiency. This result was consistent with that reported by Manios, et al. [36], where it was established that vitamin D-sufficient individuals spend less time sitting, more time in outdoor activities, and more time in physical activity compared to 25(OH)D-deficient individuals. One explanation of 25(OH)D sufficiency in physically active patients is greater exposure to UV-B rays from the sun, which, as aforementioned, contributes to vitamin D synthesis in the skin [37,38]. According to Holick [11], exposure of arms and legs between 10:00 a.m. and 3:00 p.m. for up to 30 min twice a week is sufficient to achieve adequate vitamin D levels. However, studies have reported that physical activity increases the concentration of 25(OH)D regardless of sun exposure [37,39,40,41,42]. A possible explanation is because the muscle mass stores more amounts of 25(OH)D [41]. It has also been proposed that growth hormone stimulation in response to physical activity contributes to the production of insulin-like growth factor 1, which may influence hepatic vitamin D metabolism [42].

Beyond the association of the food intake quality and intense physical activity with 25(OH)D sufficiency identified in our report, according to an ecological study by Greene, et al. [43], adherence to a healthy dietary pattern is associated with fewer COVID-19 cases and deaths. In particular, the previously mentioned study analyzed adherence to the Mediterranean diet (rich in fresh vegetables and fruits, whole grains, nuts, fish, and extra virgin olive oil) where well-being factors such as income, education, and life satisfaction increased the strength of the association [43,44]. Greene, et al. suggest that this result is due to the anti-inflammatory and chronic-disease reducing effects related to the intake of foods included in the Mediterranean diet [43]. Likewise, Tavakol, et al. [45] reported that patients with a healthy diet pattern presented a lower severity of COVID-19. This has been consistent with other studies on respiratory infections and risk of mortality from respiratory diseases [46,47]. In addition, Tavakol, et al. [45] also found that physical activity is associated with reduced severity of COVID-19, since physical exercise can enhance the immune response by reducing the influx of inflammatory cells, chemokines and proinflammatory cytokines into the lungs. Therefore, a healthy lifestyle characterized by eating healthy foods and engaging in physical activity in addition to improving 25(OH)D concentration may also predict a better course of respiratory diseases.

In addition to vitamin D, other micronutrients have also been reported to be involved in the immune response [48]. In the present study, apart from insufficient vitamin D intake, it was also identified that the assessed patients positive for SARS-CoV-2 had insufficient intakes of vitamin E, calcium, iron, and zinc, based on the RDA’s for the Mexican population. According to a study reported by Vogel-Gonzalez, et al. [49], COVID-19 patients with a serum zinc concentration <50 μg/dL presented a higher inflammatory state and greater clinical severity of the disease. In regard to zinc status, in vitro studies have reported that zinc ions play a role in the antiviral immune response by inducing the production of interferons (IFN-α and IFN-γ) [50]. It has also been shown that zinc can inhibit viral replication by interfering with the activity of RNA-dependent RNA polymerase, an enzyme in RNA viruses that is necessary for their replication [50]. Therefore, because the human body does not have a reservoir of this essential trace element, adequate dietary intake or supplementation is crucial. In regard to calcium, it is necessary as a second messenger in the proliferation, activation, and differentiation of lymphocytes [51]. In turn, vitamin E and iron, it has been reported that their functions in the immune system consist of protecting cell membranes from free radicals and the generation of reactive oxygen species by neutrophils for the neutralization of pathogens, respectively [48].

Although the evidence for the role of vitamin C in COVID-19 is weak, its use as an adjuvant treatment has been suggested. The activity of this vitamin in the immune system is related to the stimulation of phagocyte production and its potent antioxidant activity [52]. In the case of vitamin A, it is essential for the homeostasis and integrity of epithelial mucous membranes, although it has also been proposed to play an important role in the production of neutralizing antibodies, particularly in the promotion of secretory IgA against pathogens in the respiratory tract [53,54,55]. Likewise, folic acid is related to the production of antibodies [48,52]. In our study, the vitamin A, C, and folic acid intakes of the evaluated individuals were not different from the RDA’s. Finally, it has been reported that vitamins B6 and B12 are necessary for the cytotoxic activity of NK cells [48,56]. The intake of both vitamins in the evaluated individuals was higher than the RDA’s for the Mexican population; however, no individual exceeded the upper intake levels.

In addition to the Mexican population, other populations in developing and developed countries have insufficient micronutrient intakes [12,57,58,59]. Although this is a worldwide public health problem, the containment measures adopted during the COVID-19 pandemic have changed people’s diet and physical activity, such that insufficient intake of vitamins and minerals, as well as sedentary lifestyles, may have increased [13,14]. Therefore, due to the importance of micronutrients in the immune system and the benefits of physical activity, ministries of health should continue to promote the importance of a healthy lifestyle, especially in those countries with low immunization rates and high rates of COVID-19 infection and mortality.

Eating at least five servings of vegetables and fruits per day provides an important amount of vitamins and minerals [5]; however, zinc, vitamin C, and vitamin D are the micronutrients that have shown the best results on immune function [60]. Daily intake of lentils, beans, broccoli, spinach, cauliflower, pumpkin, oranges, grapes, and strawberries, in addition to some animal sources such as meat, fish, eggs, and dairy products, is recommended, as these are foods rich in these micronutrients [5].

Since RDAs are designed based on an energy intake of 2000 kcal per day in a healthy population, it is important for health professionals to establish nutritional requirements based on the individual characteristics of patients [5]. It has even been proposed that, in order to optimize the immune response, micronutrient RDAs can be exceeded through supplementation as long as care is taken not to exceed the upper intake limits. Although it is uncertain whether supplementation prevents COVID-19, this strategy should be considered in some cases for the restoration of serum concentrations, particularly in populations at high risk of insufficient micronutrient intake [5,61]. In the case of zinc, healthy individuals should meet the RDA, while in infected patients, doses of up to 75 mg of zinc through supplements in fractions of six to eight doses every 2 to 3 h are recommended. On the other hand, the recommended intake of vitamin C can be increased up to 200 mg per day in healthy subjects and up to 1 to 2 g per day in infected individuals [61]. As for vitamin D, it has been proposed that the optimal serum concentration of 25(OH)D is 40 to 60 ng/mL for the prevention of respiratory infections. For this, under the supervision of a professional, supplementation with 250 μg of vitamin D_3_ for a few weeks and then 125 μg for the maintenance of the recommended serum concentration is necessary [62]. According to the Endocrine Society, the vitamin D recommendation in healthy individuals for a serum concentration of 30 ng/mL 25(OH)D should not exceed 50 μg of vitamin D_3_ per day [17]. As mentioned above, the sun’s rays are the main source of this vitamin and sun exposure up to skin erythema can provide an equivalent of 500 μg of ingested vitamin D; however, sun exposure that exceeds more than 30 min per day is not recommended [11].

In the present study, physical activity was associated with 25(OH)D sufficiency; nevertheless, it may also improve immune response, mental health, and combat unhealthy lifestyles [63,64]. The general physical activity recommendation for adults is a minimum of 150 min of moderate physical activity per week, or 60 min of intense physical activity per week, either walking, dancing, or walking up and down stairs. In addition, strength exercises are recommended at least two days a week using one’s own body weight through squats, elbow bends, and lunges [63].

To the best of our knowledge, this is the first study to evaluate dietary intake of nutrients in SARS-CoV-2 positive patients in the Mexican population. In addition, not only the concentration of 25(OH)D was analyzed, but also factors associated with its deficiency in newly diagnosed subjects. This is also important since most studies have evaluated 25(OH)D concentration in hospitalized patients, who may be deficient as a result of inflammation.

However, our study has some limitations. First, the sample size is small, and the inclusion of more individuals in the study could have increased the variability of serum 25(OH)D concentration. Second, dietary intake was assessed with a 24-h dietary recall; although this tool is frequently used in studies of this type and gives an approximation of habitual nutrient intake, it does not reflect intake over a long period of time. In addition, the symptomatology of COVID-19, such as fatigue, nausea, diarrhea, ageusia, and anosmia, cause loss of appetite and changes in the perception of food flavors. Therefore, these symptoms could have caused underreporting of habitual dietary intake in the 24-h dietary recalls. Third, individuals with obesity were not excluded; this variable could have an effect on serum 25(OH)D concentration since it is an important factor in vitamin D deficiency. Fourth, there was no control group, so it is impossible to determine whether 25(OH)D concentration is different from healthy subjects and whether the predictor variables have a different association. Finally, these patients were not followed up, therefore, it is not possible to establish causality between the analyzed variables.

## 5. Conclusions

In conclusion, a higher level of food intake quality and minutes of intense physical activity per day are associated with 25(OH)D sufficiency in early-diagnosed SARS-CoV-2 positive patients from Mexico. However, more robust and follow-up studies are needed to determine which food groups associated with adequate diet quality have the most significant effect on 25(OH)D concentrations. We also believe that it is important to conduct studies to determine the mechanisms by which physical activity can improve serum vitamin D concentration. Finally, we believe it is necessary to pay attention not only to vitamin D, but also to other micronutrients with immunomodulatory effects that are often deficient in developing populations.

## Figures and Tables

**Table 1:** Clinical features of SARS-CoV-2 positive patients.

Variable	Total *n* = 40
Female, *n* (%)	24 (60)
Age (years) ^1^	43.98 ± 13.65
BMI (kg/m^2^) ^1^	26.4 ± 5.26
BMI classification, *n* (%)	
Normal weight	16 (40)
Overweight	16 (40)
Obesity	8 (20)
Waist (cm) ^1^	91.02 ± 14.07
Central obesity, *n* (%)	
Female (≥88 cm)	11 (45.8)
Male (≥102 cm)	5 (31.3)
Systolic (mmHg) ^1^	111.6 ± 18.33
Diastolic (mmHg) ^1^	74.7 ± 14.1
Oxygen saturation (SpO_2_%) ^2^	93 (86.1–97)
Heart rate (bpm) ^1^	79.6 ± 12.37
Temperature (°C) ^1^	36.8 ± 0.34
Respiratory rate (BPM) ^2^	20 (15.05–30)
Transferrin (ng/mL) ^2^	236 (178.25–365.45)
Ferritin (ng/mL) ^2^	138.5 (8.64–533.75)
D-dimer (ng/mL) ^2^	294.45 (129.81–1229.83)
25(OH)D (ng/mL) ^2^	22.7 (12.71–45.24)
Comorbidity, *n* (%)	12 (30)
Moderate physical activity (min/d) ^2^	30 (0–360)
Intense physical activity (min/d) ^2^	0 (0–150)
Food intake quality, *n* (%)	
Healthy Food Intake	15 (37.5)
Habits in Need of Improvement	12 (30)
Unhealthy Food Intake	13 (32.5)
COVID-19 Symptoms, *n* (%)	38 (95)

^1^ Mean ± standard deviation; ^2^ Median (percentile: p5th–p95th); BMI, Body mass index.

**Table 2:** Dietary micronutrient intake and comparison with RDA’s for the Mexican population.

Variable	Micronutrient Intake ^1^ *n* = 40	RDA’s for Mexican Population	*p*-Value	Percentage of Adequacy ^2^
Vitamin A (μg RE)	508.35 ± 238.62	568	0.122	89.5 (76.06–102.94)
Vitamin D (μg)	3.85 ± 5.03	10	**<0.0001**	38.5 (22.41–54.6)
Vitamin E (mg)	5.11 ± 3.42	11	**<0.0001**	46.45 (36.5–56.41)
Vitamin K (μg)	56.95 ± 74.37	78	0.081	73 (42.52–103.51)
Vitamin C (mg)	80 ± 71.02	60	0.083	133.34 (95.48–171.19)
Thiamin (mg)	0.91 ± 0.5	0.8	0.190	113.25 (93.18–133.32)
Riboflavin (mg)	1.23 ± 0.61	0.84	**<0.001**	146.49 (123.18–169.8)
Niacin (mg)	14.61 ± 7.33	11	**0.003**	132.84 (111.55–154.14)
Pantothenic acid (mg)	2.94 ± 1.73	4	**<0.001**	73.61 (59.78–87.45)
Vitamin B6 (mg)	1.68 ± 1.94	0.93	**0.018**	181.16 (114.51–247.81)
Folic acid (μg)	498.53 ± 928.16	380	0.424	131.19 (53.08–209.31)
Vitamin B12 (μg)	4.12 ± 6.26	2.1	**0.048**	196.09 (100.69–291.49)
Calcium (mg)	644.46 ± 226.73	900	**<0.0001**	71.61 (63.55–79.66)
Iron (mg)	10.48 ± 5.14	17	**<0.0001**	61.62 (51.96–71.29)
Zinc (mg)	7.49 ± 3.05	10	**<0.0001**	74.92 (65.15–84.69)

Micronutrient intake were adjusted for total energy using the residual method; ^1^ Mean ± standard deviation, Student’s *t*-test; ^2^ Mean (95% confidence interval for mean); RDA, Recommended Dietary Allowance; Significant *p*-values are highlighted in bold.

**Table 3:** Comparison between patients with 25(OH)D insufficiency and sufficiency.

Variable	25(OH)D Insufficiency <29 ng/mL *n* = 32	25(OH)D Sufficiency >30 ng/mL *n* = 8	*p*-Value
BMI (kg/m^2^) ^1^	26.35 ± 4.74	26.58 ± 7.37	0.914
Waist (cm) ^1^	92.18 ± 12.85	86.38 ± 18.46	0.302
Food intake quality, *n* (%) ^2^			
Unhealthy Food Intake	13 (40.6)	0 (0)	
Habits in Need of Improvement	10 (31.3)	2 (25)	
Healthy Food Intake	9 (28.1)	6 (75)	**0.029**
Fitzpatrick skin phototype, *n* (%) ^2^			
Type I	0 (0)	0 (0)	0.883
Type II	6 (18.8)	1 (12.5)	
Type III	13 (40.6)	3 (37.5)	
Type IV	12 (37.5)	4 (50)	
Type V	1 (3.1)	0 (0)	
Type VI	0 (0)	0 (0)	
Sun exposure (min/d) ^3^	25 (0–243)	120 (15–180)	**0.017**
Sun exposure (10 a.m.–3 *p*.m.), *n* (%) ^4^	10 (31.3)	6 (75)	0.154
Sunscreen, *n* (%) ^4^	16 (50)	2 (25)	0.193
Vitamin D (μg) ^3^	2.44 (0–13.81)	2.39 (0–12.14)	0.919
Comorbidity, *n* (%) ^4^	10 (31.3)	2 (25)	0.548
Moderate physical activity (min/d) ^3^	30 (0–402)	45 (0–105)	0.390
Intense physical activity (min/d) ^3^	0 (0–101.25)	80 (0–142.5)	**0.032**
COVID-19 Symptoms, *n* (%) ^4^	32 (100)	6 (75)	**0.036**

^1^ Mean ± standard deviation, Student’s *t*-test; ^2^ Percentage, *χ^2^* test; ^3^ Median (percentile: p5th–p95th), Mann-Whitney *U* test; ^4^ Percentage, Fisher’s exact test; BMI, Body mass index; Fitzpatrick skin phototype: Type I–Always burns, never tans; Type II–Always burns, tans with difficulty; Type III–Sometimes mild burn, tan about average; Type IV–Rarely burns, tans easily; Type V–Never burns, tans easily; Type VI–Never burns, tans very easily; Significant *p*-values are highlighted in bold.

**Table 4:** Association analysis of 25(OH)D insufficiency vs. sufficiency.

Univariate Analysis			
**Variable**	**OR**	**95% CI**	***p*-value**
Food intake quality	5.51	1.27–24.02	**0.023**
Intense physical activity (min/d)	1.02	1.01–1.04	**0.011**
Sun exposure (min/d)	1.01	0.99–1.02	0.069
Vitamin D intake (μg)	1.16	0.99–1.35	0.054
**Multivariate Analysis**			
**Variable**	**OR**	**95% CI**	***p*-value**
Food intake quality ^1^	6.79	1.18–39.09	**0.032**
Intense physical activity (min/d) ^2^	1.04	1.01–1.07	**0.013**

Multivariate analysis adjusted for age, weight, BMI, and presence of comorbidity; Odds ratio 95% confidence interval; ^1^ R-squared for the predictive model: *R^2^* = 33.4%, *p* = 0.09; ^2^ R-squared for the predictive model: *R^2^* = 42.1%, *p* = 0.03; Significant *p*-values are highlighted in bold.

## Data Availability

The data presented in this study are not publicly available.
